# Complete Absence of Iliac Arteries in the Left Hemipelvis in a Case of Deceased Donor Renal Transplantation

**DOI:** 10.1155/2015/138170

**Published:** 2015-04-30

**Authors:** Ebrahim Palkhi, Samir Pathak, Lutz Hostert, Gareth Morris-Stiff, Jai V. Patel, Niaz Ahmad

**Affiliations:** ^1^Division of Surgery, Department of Transplantation, St James's University Hospital, Leeds LS9 7TF, UK; ^2^Department of Vascular Radiology, St James's University Hospital, Leeds LS9 7TF, UK

## Abstract

Renal transplantation is an established method of treating end-stage renal failure. Whilst the majority of procedures follow a standard technique, vascular anomalies may pose intraoperative challenges and, therefore, careful preoperative assessment is warranted. We present a unique, complex case compounded by complete absence of iliac arteries in the left hemipelvis in association with double inferior vena cava in a young recipient.

## 1. Introduction

Conventional renal transplantation is performed with placement of the kidney in the iliac fossa, vascular anastomoses between the donor renal and recipient iliac vessels, and ureteric anastomosis to the bladder [1, 2]. Difficulties and challenges may arise due to abnormalities of the iliac vessels or of the lower urinary tract [2]. The most common vascular anomalies are acquired and include thrombosis of the external/common iliac vein and/or the inferior vena cava (IVC) due to previous venous catheter placement for dialysis and atherosclerotic stenosis or occlusion of the iliac arteries [3]. Congenital vascular abnormalities with absent pelvic vessels are extremely rare in the context of renal transplantation [3, 4]. We present a case of complete absence of the iliac arteries in the left hemipelvis in association with a double inferior vena cava.

## 2. Case Report

A 28-year-old female was admitted for deceased donor renal transplantation (DDRT). Her end-stage renal failure was secondary to reflux nephropathy. She had a previous deceased donor renal transplant at the age of five that had failed after 16 years. The initial transplant was straightforward and no difficulties were encountered with respect to recipient vascular anatomy (arterial and venous anastomoses were to the aorta and IVC, resp.). She was established on haemodialysis and had been on the waiting list for seven years. In addition to her renal disease, the patient had adolescent onset severe kyphoscoliosis and had undergone surgical reconstruction for vaginal atresia.

The donor was a 36-year-old male, brain stem dead donor, who had died secondary to a hypoxic brain injury. The right kidney was allocated and had a single renal vein and two renal arteries on an aortic patch. The HLA mismatch to the donor was 2A, 2B, and 1DR.

**Figure 1:**
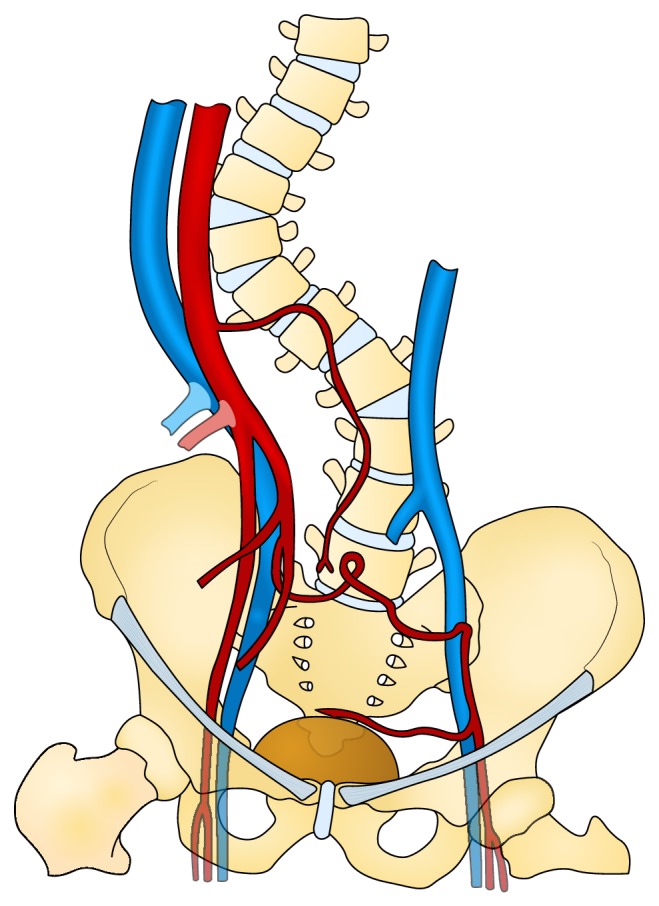
Kyphoscoliosis and relationship to vessels.

Preoperatively there was difficulty in identification and intubation of the external urethral meatus. The placement of the urinary catheter was confirmed with an on-table cystogram. A standard left iliac fossa Gibson incision and an extraperitoneal approach were used for implantation. The left external iliac artery was absent. The left external iliac vein was patent and of normal calibre. There was a tortuous collateral artery 2-3 mm in diameter running across the pelvis to the left of the external iliac vein just above the inguinal ligament. This then divided with a branch continuing under the inguinal ligament and another branch running across to the surface of the bladder. A normal pulse was palpable in the left common femoral artery just below the inguinal ligament. Upon further proximal dissection, no internal iliac or common iliac artery could be identified. The external iliac vein appeared to be draining into a left sided inferior vena cava (Figure 1). Further extension of the dissection to explore the right-sided anatomy was not deemed safe due to the patient's body habitus.

**Figure 2:**
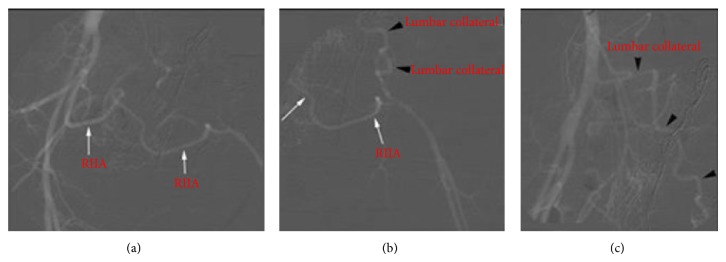
Angiogram demonstrating the anatomy of the aorta and iliac arteries. Patent aorta, right common, internal, and external iliac arteries with hypertrophied collateral arising from the right internal iliac artery (RIIA) coursing from the right to the left side (white arrows) and a hypertrophied L5 lumbar artery collateral arising from the aorta (black arrowheads) reconstituting the left femoral artery.

An on-table angiogram was performed to map the recipient arterial and venous anatomy. This confirmed that the abdominal aorta was located on the right with only a single right iliac system and absence of the left iliac system. The common femoral artery on the left was formed from a hypertrophied lumbar artery and further collaterals from the right internal iliac artery in the pelvis (Figure 2). A venogram confirmed the presence of a duplex inferior vena cava. Following angiographic mapping of the vascular anatomy, the left iliac fossa wound was closed and a midline laparotomy was performed. The aorta and right IVC were isolated above the site of the previous transplant. Unfortunately, when the kidney was removed from cold storage it was found to be frozen and transplantation had to be abandoned.

**Figure 3:**
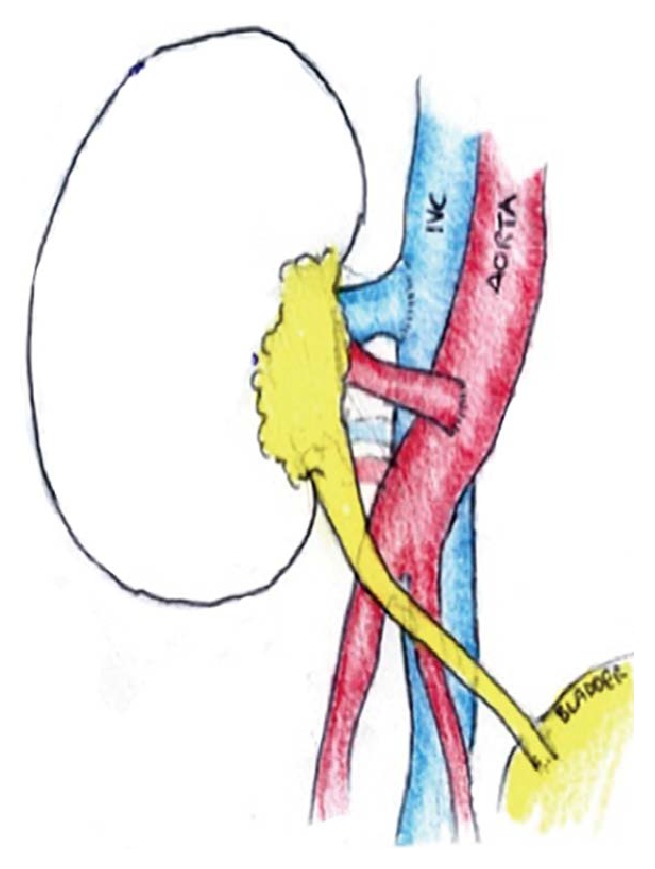
Schematic diagram of the implantation of kidney with vascular anastomosis to distal aorta and inferior vena cava (IVC).

In view of the difficult anatomy, extensive dissection, and difficult match-ability of the recipient, a compassionate allocation of a deceased donor kidney was sought from the national allocation scheme. Three days later a kidney was allocated to the recipient from a 57-year-old male brain dead donor with an HLA mismatch of 0A, 1B, and 1DR. This was successfully implanted through the previous laparotomy incision onto the aorta and the right IVC (Figure 3). The patient experienced delayed graft function (DGF) and was discharged home on day 20 after establishing normal renal function.

## 3. Discussion

The surgical approach to the adult kidney transplantation has not changed significantly since the first description of the procedure in 1951 [5]. Kuss and colleagues reported using a pelvic approach whereby the donor graft is transplanted into the extraperitoneal space, with vascular anastomoses to the iliac vessels [5]. The right side is often preferred because the vessels are more superficial and operating on the right is more convenient for the right-handed surgeon. Depending upon the anatomy of the donor kidney, recipient vessels, and preference of the surgeon, the arterial anastomosis can be performed to the external iliac, internal iliac, or common iliac artery. The venous anastomosis is usually to the external iliac vein. After reperfusion of the kidney, the ureter is anastomosed to the dome of the bladder, with or without an antireflux mechanism, over a JJ ureteric stent [2, 6].

An assessment of the pelvic vasculature is important prior to listing patients for renal transplantation although the protocol for assessment is variable among centres. In all cases a clinical assessment of the femoral artery and distal vessels is mandatory [7]. In older patients and those with a history of diabetes, smoking, peripheral vascular disease, and femoral arterial or venous cannulation and individuals with a previous transplant, a more formal assessment with imaging is required. This is usually in the form of a duplex ultrasound scan, magnetic resonance angiogram (MRA), or computerised tomography angiogram (CTA) [7, 8].

An assessment of pelvic anatomy, including the blood vessels, is also important in recipients with congenital pelvic anomalies because of their spatial association with other anomalies. Our patient was born with congenital vaginal atresia and had developed severe kyphoscoliosis during her adolescence. She also had a previous transplant as a child. Nevertheless she was young and had strong femoral and distal pulses bilaterally. Therefore, it was felt that a formal assessment with imaging was not essential. Her previous transplant was as a child and was on the right side with vascular anastomoses to the IVC and the aorta. This would not have raised suspicion of the anomalous left iliac arterial system.

Congenital abnormalities of the iliac and femoral vessels are rare findings; the most reported findings include iliofemoral aplasia associated with persistent sciatic artery or atresia with residual cord [3, 9, 10]. The persistent sciatic artery supplies blood to the lower limb bud during embryological development and normally regresses and is replaced by the iliac and femoral vessels. Failure to regress leads to a persistent sciatic artery. In cases of incomplete regression, the persistent sciatic artery becomes hypoplastic and the external iliac system is absent; therefore the arterial supply to the lower limb develops through collaterals [3]. Our case may represent an incomplete regression of the sciatic artery with unilateral absence of the iliac system [11, 12]. The common femoral artery below the inguinal ligament on the affected side was normal and was filled by collaterals from hypertrophied lumbar arteries and a collateral artery from the right internal iliac artery (Figures 1 and 2).

Implantation of the kidney had to be abandoned once the vascular anatomy was mapped out as the donor kidney was “frozen” when removed from cold storage. The preservation solution was scanty (200–300 mL) and there were small lumps of ice in direct contact with the kidney. Such an incidence has never been encountered in our centre in 4000 renal transplants. The kidney had been procured and stored using standard protocol [13]. The kidney was flushed in situ and preserved using hyperosmolar citrate preservation solution. The back-table preparation was performed in the recipient centre, and the surgeon reported nothing untoward. It is likely that small blocks of “saline ice” were placed with the preservation solution. Frozen saline is normally used to maintain a cold bed for bench dissection [14]. Subsequent analysis of the preservation solution suggested a likely contamination with normal saline.

## 4. Conclusions

Congenital absence of the iliac arterial system in a hemipelvis is extremely rare and has not been reported in the renal transplant literature previously. Given the history of congenital vaginal atresia, adolescent onset kyphoscoliosis, and previous transplant in our recipient, a formal assessment should have been carried out and we propose that all renal transplant recipients, especially with congenital anomalies, warrant a formal assessment of vascular anatomy, via radiological imaging even if clinical assessment appears to be satisfactory.

## Abbreviations

IVC: Inferior vena cava
HLA: Human leucocyte antigen
DGF: Delayed graft function
DDRT: Deceased donor renal transplant
MRA: Magnetic resonance angiography
CTA: Computed tomography angiography.
